# A novel hypoxia-driven gene signature that can predict the prognosis of hepatocellular carcinoma

**DOI:** 10.1080/21655979.2022.2073943

**Published:** 2022-05-13

**Authors:** Zhirui Zeng, Shan Lei, Jingya Wang, Yushi Yang, Jinzhi Lan, Qianting Tian, Tengxiang Chen, Xiaojiang Hao

**Affiliations:** a Guizhou Provincial Key Laboratory of Pathogenesis & Drug Research on Common Chronic Diseases, Department of Physiology, School of Basic Medical Sciences, Guizhou Medical University, Guiyang, China; b State Key Laboratory of Functions and Applications of Medicinal Plants, Guizhou Medical University, Guiyang, China; c Key Laboratory of Chemistry for Natural Products of Guizhou Province, Chinese Academy of Sciences, Guiyang, China; d Department of Pathology, Affiliated Hospital of Guizhou Medical University, Guiyang, China; e Precision Medicine Research Institute of Guizhou Medical University, Affiliated Hospital of Guizhou Medical University, Guiyang, China

**Keywords:** HCC, Hypoxia-driven gene, prognostic prediction, CFHR3

## Abstract

Hypoxia environment exists in already started hepatocellular carcinoma (HCC) and promotes its progression by driving changes in the gene expression profiles of cells. However, the status of hypoxia-driven genes in HCC is largely unknown. In the present study, 368 HCC tissues from The Cancer Genome Atlas were divided into high and low hypoxia groups according to their hypoxia signatures. A total of 1,142 differentially expressed genes (DEGs) were identified between the two groups, and 34 of these DEGs were highly expressed in HCC tissues compared with adjacent tissues, especially in HCC tissues from patients with stage III–IV HCC. After constructing a protein–protein interaction network and applying the least absolute shrinkage and selection operator Cox regression method for 34 DEGs, a three-gene signature (complement factor H related 3 [*CFHR3*], egl-9 family hypoxia inducible factor 3 [*EGLN3*], and chromogranin A [*CHGA*]) was constructed and had prognostic value to predicted outcome of patients with HCC. This three-gene signature was suitable for classifying patients with HCC in the International Cancer Genome Consortium. CFHR3 shows remarkable diagnostic value in HCC. Hypoxia decreased CFHR3 expression, but increased HCC cell proliferation and motility. Overexpression of CFHR3 in HCC cells under hypoxia reversed the stimulatory effects of hypoxia and suppressed cell proliferation and metastasis *in vivo*. In conclusion, we identified a novel hypoxia-driven gene signature (*CFHR3, EGLN3*, and *CHGA*) for reliable prognostic prediction of HCC, and demonstrated that overexpression of CFHR3 may be a potential strategy to overcome hypoxia and treat HCC.

## Graphical abstract

**Figure:**
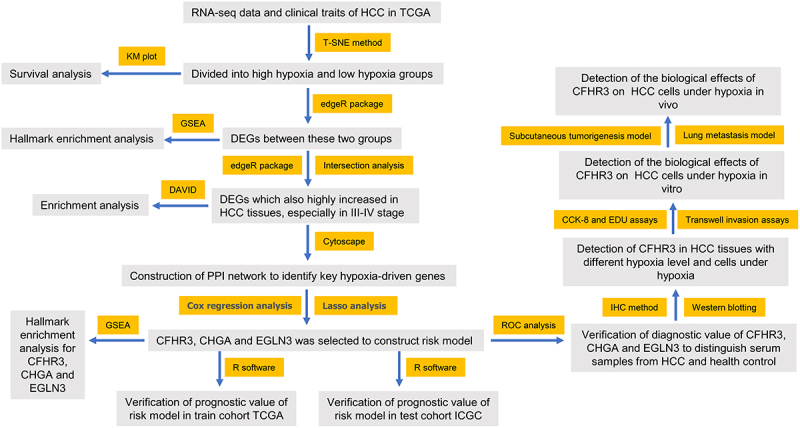

## Research highlights

- *CFHR3, EGLN3*, and *CHGA* were used to construct a hypoxia-driven gene signature.
- The hypoxia-driven gene signature is a reliable prognostic predictor for HCC.
- *CFHR3* has diagnostic value for HCC.
- Overexpression of *CFHR3* blocks the effects of hypoxia.

## Introduction

Hepatocellular carcinoma (HCC) is a common malignant tumor of the digestive system and the third leading cause of cancer-related deaths worldwide [1]. Due to the high rates of metastasis and recurrence of HCC and its innate drug resistance, the five-year survival rate of patients with HCC remains unsatisfactory even with targeted therapy [2,3]. Alpha-fetoprotein (AFP) is a recognized diagnostic marker for liver cancer, but its specificity and sensitivity are poor when used alone [4]. Therefore, it is essential to identify novel diagnostic markers and therapeutic targets for HCC treatment.

Hypoxia is an important characteristic of solid tumors. During the progression of solid tumors, hypoxic areas spontaneously form in the interior of solid tumors due to the development of a malformed vascular system [5,6]. A hypoxic microenvironment is important for mediating the increase in proto-oncogene expression levels in HCC cells and for promoting angiogenesis, cell metastasis, and proliferation [7]. Hypoxia-inducible factors (HIFs) are the most direct response elements in cells that respond to hypoxia and can bind to a series of gene promoters to promote gene transcription [8]. A series of target genes of HIFs, including *PLAG1* like zinc finger 2 [9] and ubiquitin-specific peptidase 22, have been identified [10]. As a hypoxia-driven gene, the expression levels of vasodilator stimulated phosphoprotein are elevated in HCC tissues, and its overexpression is positively associated with poor prognosis of patients [11]. The expression of peroxisome proliferator activated receptor gamma, a hypoxia-driven gene expressed in HCC, is significantly decreased under hypoxia, whereas its deficiency induces cell resistance to sorafenib [12]. However, most hypoxia-driven genes that are directly or indirectly affected by HIFs and their status in HCC remain unknown. Therefore, the identification of hypoxia-driven genes in HCC cells will aid in uncovering the hypoxia regulatory network and provide novel and effective therapeutic targets to overcome the malignant behavior of HCC cells mediated by the hypoxic microenvironment.

The present study aimed to identify hypoxia-driven genes of HCC, establish a risk model that can aid in the prognosis prediction of patients with HCC, and explore effective targets to overcome the stimulatory effects of hypoxia in HCC.

## Materials and methods

### Data acquisition and preprocessing

The RNA-seq profiles and corresponding clinical characteristics of HCC in The Cancer Genome Atlas (TCGA) [13] were downloaded from https://portal.gdc. cancer. gov and set as the training cohort, which included a total of 368 HCC tissues and 50 adjacent tissues. The gene expression profiles of 232 HCC tissues and the corresponding clinical characteristics obtained from the International Cancer Genome Consortium (ICGC) (https://dcc.icgc.org) [14] were used as the test cohort. R software (version 4.1.2) was used to convert Ensemble IDs to gene symbols and perform data normalization prior to subsequent analysis.

### Identification of hypoxic characteristics in HCC tissues

To assess the hypoxia level of HCC tissues, we first obtained the names of 50 genes listed with the term of hypoxia (BUFFA_HYPOXIA_METAGENE) from the Gene Set Enrichment Analysis (GSEA) database (http://www.gsea-msigdb.org/gsea/index.jsp) [15]. The expression levels of these 50 genes in HCC tissues were extracted, and the t-distributed stochastic neighbor embedding (t-SNE) method [16] was used to calculate the characteristic values of the 50 hypoxia genes in each sample. We used these characteristic values to represent the hypoxia level of HCC tissues; therefore, HCC tissues in TCGA database were divided into high and low hypoxia groups according to

median characteristic values. The grouping conditions are listed in Supplementary Table S1.

### Survival analysis

Differences in overall survival rates between patients with HCC in the high hypoxia group and those in the low hypoxia group were analyzed using SPSS 19.0. Statistical significance was set at P < 0.05.

### Analysis of differentially expressed genes (DEGs)

DEGs between the two groups (high hypoxia *vs* low hypoxia, HCC tissues *vs* adjacent tissues, and stages III–IV *vs* stages I–II) were analyzed using R software with the EdgeR package [17]. The threshold to distinguish DEGs was set as |LogFC>1| and adjusted P < 0.05. All genes with LogFC and adjusted P values are shown in volcano plots, while DEGs are shown in heatmaps.

### GSEA analysis

Patients were divided into high and low groups (top 50%, high *vs* bottom 50%, low) and analyzed using GSEA software (version 4.0.3) to determine whether the pre-defined hallmark pathways were enriched. After calculating the normalized enrichment scores (NES) and adjusted P values, the hallmark pathway terms with P < 0.05 were set as significant and the significant terms with the top five NES were visualized.

### Biological process enrichment analysis

Genes were imported into a database for annotation, visualization, and integrated discovery (DAVID) (https://david.ncifcrf.gov/) [18]. Biological process terms with P < 0.05 were set as significant and were visualized using R software.

### Protein–protein internetwork (PPI) construction

Genes were imported into the STRING database (https://cn.string-db.org/) [19] to construct a preliminary PPI network with reliability >0.4. Discrete nodes are removed. Information on the preliminary PPI network was exported and visualized using Cytoscape software (version 3.9.1) [20].

### Prognostic gene screening and prognostic signature

Relationships between gene expression patterns and overall survival were analyzed using R software with univariate COX regression analysis. P < 0.05 was set as the threshold to screen candidate prognostic genes. The overlapping candidate prognostic genes from the two analyses were further screened using least absolute shrinkage and selection operator (LASSO) Cox regression analysis for variable selection and shrinkage using the glmnet package (version 3.0) [21]. Based on the coefficients of the risk model, a formula was developed to calculate the prognostic risk score of each patient. The patients were then divided into high -and low-risk groups based on the median risk score. Kaplan-Meier survival analyses were performed for the two groups to assess the clinical value of the risk model, and receiver operating characteristic (ROC) curves were used to evaluate the stability of the model.

### Serum specimen analysis

The gene expression profile GSE112679 [22] contained 1,204 serum specimens from patients with HCC and 958 serum specimens from healthy individuals, and was downloaded from the Gene Expression Omnibus (GEO) database (https://www.ncbi.nlm.nih.gov/gds). After merging, normalization, and gene annotation, the expression of the target genes was extracted. The diagnostic value of the target genes was analyzed using an ROC curve based on their expression. AUC > 0.7 and P < 0.05 were used as cutoff values to determine whether the target genes had a distinctive diagnostic value.

### Clinical sample collection

HCC tissues of the patients enrolled in the present study were obtained from the Affiliated Hospital of Guizhou Medical University. The enrolled patients did not undergo chemoradiotherapy before sample collection. The exclusion criteria were as follows: (1) multiple tumors; (2) HCC was not the primary lesion; (3) comorbidities in the hematologic system; and (4) refusal to participate. Finally, 40 HCC tissues were collected and stored at – 80°C until further use. The collection and use of samples were approved by the Human Ethics Committee of Guizhou Medical University (approval number: 2021–255), and all participants provided written informed consent.

### Immunohistochemical staining

Forty HCC samples and tumor tissues were embedded in paraffin. After dewaxing and dehydration with xylene and an ethanol gradient, the tissues were immersed in 0.1 M sodium citrate solution (pH 6.0; Sigma-Aldrich, USA) and subjected to antigen retrieval using a high-temperature and high-pressure method. To reduce nonspecific binding, a solution containing 0.3% hydrogen peroxide and 5% bovine serum albumin (Servicebio, Wuhan, China) was used to block the tissues. Primary antibodies against HIF1α (1:100; Cat No. 20960-1-AP; Proteintech, Wuhan, China), carbonic anhydrase 9 (CA9; 1:200; Cat No. 11071-1-AP; Proteintech, Wuhan, China), CFHR3 (1:100; Cat No. 16583-1-AP; Proteintech, Wuhan, China), and proliferating cell nuclear antigen (PCNA; 1:200; Cat No. 10205-2-AP; Proteintech, Wuhan, China) were incubated overnight at 4°C. Following three washes with phosphate-buffered saline (PBS), the tissues were incubated with horseradish peroxidase-conjugated secondary antibodies (Boster, Wuhan, China) for 2 h and then stained with horseradish peroxidase- diaminobenzidine reagent (Beyotime, Suzhou, China). Finally, an orthotopic light microscope was used to detect antigen-antibody complex signaling in tissues.

### Cell culture and transfection

All HCC cell lines (Hep3B, Huh-7, JHH-7, Li-7, QGY-7701, SK-Hep-1, SNU-398, SNU-423, HepG2 and SMMC-7721) and normal hepatic epithelial cells (LO2) were obtained from Procell (Wuhan, China) and cultured in Dulbecco’s modified Eagle’s medium (DMEM; Biological Industries, Israel) containing 10% fetal bovine serum (FBS; Biological Industries, Israel) at 37°C with 5% CO_2_. CFHR3 plasmids and their corresponding vectors were obtained from iGene Biotechnology Co. Ltd. (Beijing, China). The normoxic environment was set as 21% O_2_, 5% CO_2_ and 74 N_2_ in a three-gas incubator, while the hypoxic environment was set as 1% O_2_, 5% CO_2_ and 94 N_2_. Transfection of plasmids and vectors was performed using Lipofectamine 2000 (Thermo Scientific, USA) for 6 h, according to the manufacturer’s protocol. To obtain stable CFHR3-overexpressing cells, they were cultured with 0.5 μg/mL puromycin for 10 d.

### Quantitative real-time fluorescence PCR (qRT-PCR)

Total RNA from HCC cells and tissues was isolated using TRIzol reagent (Invitrogen, USA). A kit of complete reagent for first-strand cDNA synthesis (Sigma-Aldrich, USA) was used to reverse transcribe mRNAs into cDNAs. The mRNA expression of target genes was determined using KAPA SYBR® FAST reagent (Sigma-Aldrich, USA). ACTB was used as a loading control. The primers used were as follows: CHGA forward primer 5ʹ-TAAAGGGGATACCGAGGTGATG-3ʹ, CHGA reverse primer 5ʹ- TCGGAGTGTCTCAAAACATTCC-3ʹ, CFHR3 forward primer 5ʹ- TGCTAATGGACAAGTGAAACCTT-3ʹ, CFHR3 reverse primer 5ʹ- GGCAACTTCTGTAGAGTTACCC-3ʹ, EGLN3 forward primer 5ʹ- CTGGGCAAATACTACGTCAAGG-3ʹ, EGLN3 reverse primer 5ʹ- GACCATCACCGTTGGGGTT-3ʹ, ACTB forward primer 5ʹ-CATGTACGTTGCTATCCAGGC-3ʹ, and ACTB reverse primer 5ʹ-CTCCTTAATGTCACGCACGAT-3ʹ.

### Western blotting

HCC cells were lysed using radioimmunoprecipitation assay buffer (Beyotime, Suzhou, China) containing 1% phenylmethylsulfonyl fluoride (Beyotime). Total proteins in HCC cells were extracted by centrifugation at 4°C and protein concentrations were determined using the BCA method. A total of 30 μg of protein from each sample was electrophoresed in sodium dodecyl sulfate-polyacrylamide gels (Meilune, Dalian, China) and then transferred to polyvinylidene fluoride membranes (Thermo Scientific, USA). Membranes were blocked using TBST containing 5% skim milk powder (Beyotime, Suzhou, China). Primary antibodies against CFHR3 (1:500; Cat No. 16583-1-AP; Proteintech, Wuhan, China), EGLN3 (1:500; Cat No. 18325-1-AP; Proteintech, Wuhan, China), CHGA (1:500; Cat No. 10529-1-AP; Proteintech, Wuhan, China) and actin beta (ACTB) (1:1000; Cat No. 20536-1-AP; Proteintech) were added and incubated overnight at 4°C. After washing three times with PBS, the membranes were incubated with secondary antibodies for 2 h and visualized using ECL reagents (Boster, Wuhan, China). The relative expression of CFHR3, EGLN3 and CHGA was normalized to that of ACTB.

### Cell proliferation assays

The viability of HepG2 and SMMC-7721 HCC cells was determined using 5-ethynyl-2 -deoxyuridine (EdU) and colony formation assays. For EdU assays, an EdU Apollo 488 Kit (Beyotime, Suzhou, China) was used according to the manufacturer’s protocol, and the EDU-positive rate in each field was recorded using an inverted fluorescence microscope (Olympus, Japan). For colony formation assays, 2,000 cells were added to each well of 6 well-plates and cultured at 37°C for 10 days. The culture medium was removed, and the cell colonies were fixed with 4% paraformaldehyde (Boster, Wuhan, China) and stained with 0.1% hematoxylin. After washing three times with PBS, the plates were photographed, and the number of colonies was counted using ImageJ software.

### Transwell assays

A total of 5 × 10^4^ HepG2 and SMMC-7721 HCC cells in 300 μL serum-free DMEM were seeded in the upper chamber of Transwell plates (bore diameter 0.8 mm; Corning, USA) that had been pre-coated with 8% Matrigel (Thermo Scientific, USA). DMEM containing 10% FBS was used as an inducer and was placed in the lower chamber. After culturing for 24 h in normoxic or hypoxic environments, the upper chambers were fixed with 4% paraformaldehyde and stained with 0.1% crystal violet. Five random fields of each upper chamber were recorded, and the number of invasive cells per field was counted using the ImageJ software (Version: 1.8.0).

### In vivo experiments

The protocols for *in vivo* experiments were approved by the Animal Ethics Committee of Guizhou Medical University (approval number 2100334, Guiyang, China). Total 32 4–6 weeks old nude mice (female; weight 18 g-20 g) were purchased from Animal Research Center of Guizhou Medical University. Mice were feed in specified pathogen free environment with 12 h light and 12 h dark. Protocol subcutaneous tumorigenesis model and pulmonary metastasis model were accordance with previous studies [23,24]. In briefly, to measure proliferation *in vivo*, HepG2 cells were resuspended in PBS, the density was adjusted to 1 × 10^7^/mL and 200 μL of each cell suspension was injected into the right flanks of mice (n = 8/group). Tumor volume was measured every 3 days. Mice were euthanized on day 24, and tumor tissues were excised from subcutaneous tissues and photographed. To detect metastasis *in vivo*, SMMC-7721 HCC cells were resuspended in PBS, the density was adjusted to 2 × 10^7^/mL and 100 μL of the cell suspension was injected into the caudal veins of mice (n = 8/group). The mice were euthanized while mice had difficulty breathing. The lung tissues of mice were extracted and metastatic foci in the lungs were detected by HE eosin staining.

### Statistical analysis

All experiments were performed in triplicate, and the results were analyzed using the SPSS software (version 19.0). Differences between groups were calculated using an unpaired Student’s *t*-test or one-way analysis of variance with Tukey’s post-hoc test. Statistical significance was set at P < 0.05.

## Results

The results of this study demonstrated that Patients with HCC with high hypoxia levels have a shorter overall survival rate. A total of 34 key hypoxia-driven DEGs had increased expression levels in HCC tissues, in patients with HCC with high hypoxia levels, and in patients with stage III–IV tumors, 27 of which interacted with each other. By performing LASSO penalized Cox regression analysis and multivariate Cox regression analysis, a risk score = (0.101109253 × chromogranin A [CHGA] expression) + (0.116251865 × egl-9 family hypoxia inducible factor 3 [EGLN3] expression) + (–0.094009858 × complement factor H related 3 [CFHR3] expression) was constructed. This risk score has a high prognostic value for HCC tissues from TCGA and ICGC. Similarly, CFHR3 also had diagnostic value in distinguishing serum samples from patients with HCC and healthy controls. CFHR3, CHGA and EGLN3 had differential expression model under hypoxia. The expression of CFHR3 was reduced in HCC tissues with high hypoxia levels in our cohort and decreased in HCC cells cultured under hypoxia. Overexpression of CFHR3 reversed the stimulatory effects of hypoxia on the proliferation and mobility of HCC cells *in vitro*. Furthermore, the overexpression of CFHR3 suppressed the proliferation and metastasis of HCC cells *in vivo*.

### Analysis of the landscape between high-hypoxia and low-hypoxia HCC tissues

**Figure 1.**
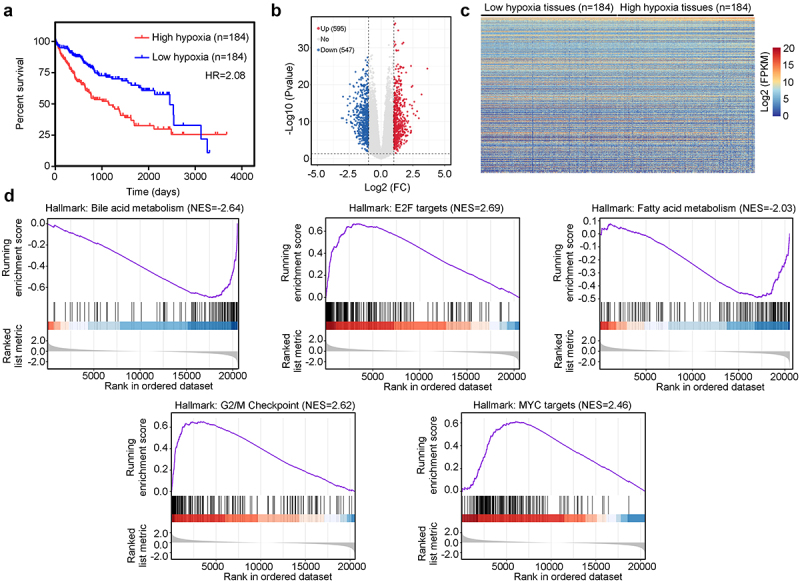
Analysis of the landscape between high- and low-hypoxia hepatocellular carcinoma (HCC). (a) Kaplan–Meier plot analysis for the overall survival of patients with high- and low-hypoxia HCC. (b) Volcano plot exhibiting changes in differentially expressed genes (DEGs) between high- and low-hypoxia HCC. (c) Heatmap plots exhibiting DEGs in high- and low-hypoxia HCC. (d) Gene set enrichment analysis (GSEA) analysis for the enrichment of hallmark terms between high- and low-hypoxia HCC.

After calculating the hypoxia signature of HCC tissues in TCGA, HCC tissues were divided into high -and low-hypoxia groups according to the median hypoxia signature score. The results of Kaplan-Meier survival analysis indicated that Patients with HCC in the high-hypoxia group had a lower overall survival rate than Patients with HCC in the low-hypoxia group (HR = 2.08, Figure 1(a)). Analysis of DEGs identified a total of 595 upregulated genes and 547 downregulated genes between high-and low-hypoxia HCC tissues (Figure 1(b,c)). GSEA analysis revealed that hypoxia in HCC was positively associated with E2F targets (NES = 2.69), G2/M checkpoint (NES = 2.62), and MYC targets (NES = 2.46), but was negatively associated with bile acid metabolism (NES = −2.64) and fatty acid metabolism (NES = −2.03) (Figure 1(d)).

### Screening of important hypoxia-driven genes in HCC tissues

**Figure 2.**
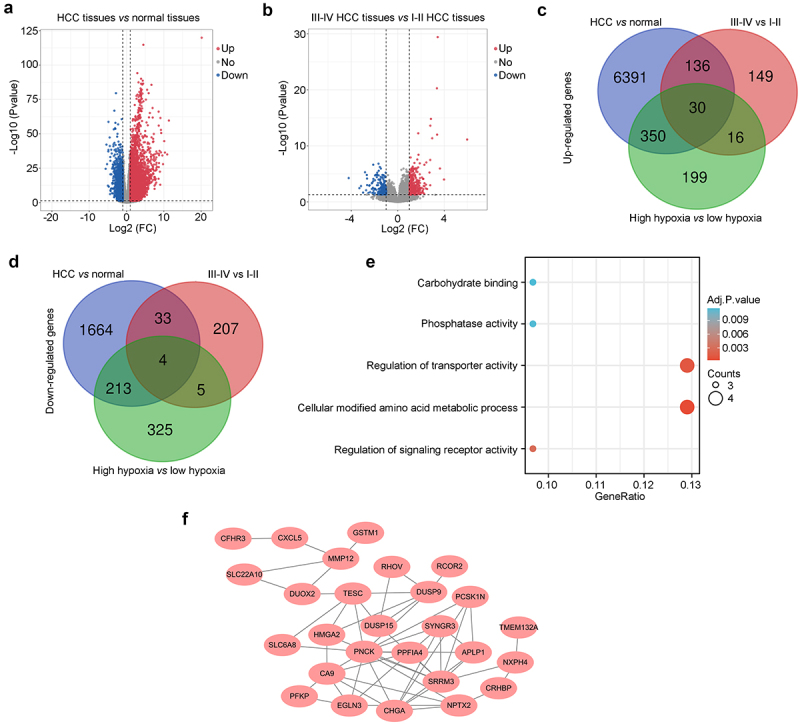
Screening of important hypoxia-driven DEGs in HCC tissues. (a) Volcano plot showing changes in DEGs between HCC tissues and adjacent non-tumor tissues. (b) Volcano plot exhibiting DEGs between HCC tissues from stage III–IV patients and stage I–II patients. (c) Exploration of DEGs that are highly expressed in HCC tissues compared with adjacent non-tumor tissues, especially in HCC with high-hypoxia levels and in patients with stage III–IV HCC. (d) Exploration of DEGs that are poorly expressed in HCC tissues compared with adjacent non-tumor tissues, especially in those with high-hypoxia levels and in patients with stage III–IV HCC. (e) Biological process analysis for the 34-candidate important hypoxia-driven DEGs. (f) Construction of a protein–protein interaction (PPI) network using the 34-candidate important hypoxia-driven DEGs, and 27 of them interacted with each other.

The aforementioned DEGs between the high-and low-hypoxia HCC tissues were identified as hypoxia-driven genes in HCC tissues. We then determined whether these hypoxia-driven genes were highly expressed in HCC tissues, particularly in patients at stages III–IV. Analysis of DEGs was performed, and a total of 6,907 upregulated DEGs and 1,914 downregulated DEGs were identified between HCC tissues and adjacent normal tissues (Figure 2(a)). Similarly, a total of 331 upregulated DEGs and 249 downregulated DEGs were identified between HCC tissues from patients at stages III–IV and stages I–II (Figure 2(b)). Intersection analysis revealed that 30 hypoxia-driven DEGs were highly expressed in HCC tissues, especially in tissues from Patients with HCC at stages III–IV (Figure 2(c)), whereas four hypoxia-driven DEGs were reduced (Figure 2(d)). Moreover, these 34 hypoxia-driven DEGs were enriched in biological process terms, including carbohydrate binding, phosphatase activity, regulation of transporter activity, cellular modified amino acid metabolic processes, and regulation of signaling receptor activity (Figure 2(e)). Furthermore, through the construction of a PPI network, we found that 27 of these 34 hypoxia-driven DEGs interacted with each other (Figure 2(f)); thus, these 27 hypoxia-driven DEGs were identified as important hypoxia-driven genes.

### Construction of a prognostic risk model in the training cohort

**Figure 3.**
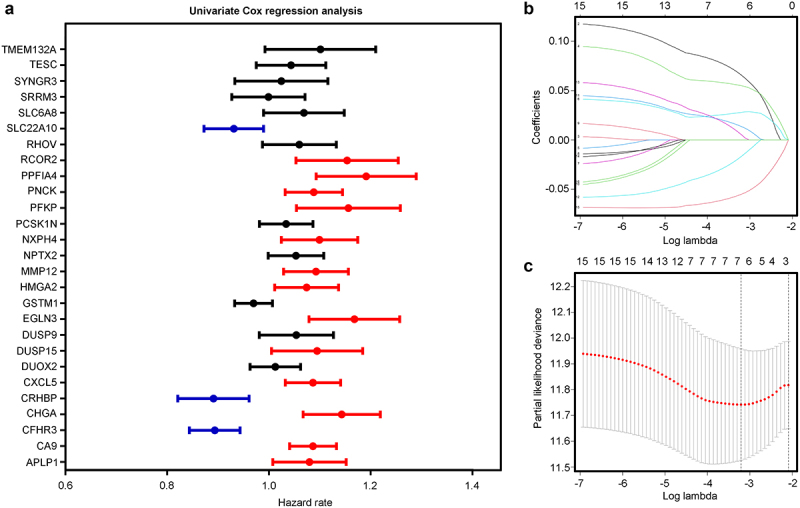
Univariate Cox regression analysis and least absolute shrinkage and selector operation (LASSO) penalized Cox regression analysis for the important hypoxia-driven DEGs in HCC tissues. (a) Univariate Cox regression analysis for the 27 important hypoxia-driven DEGs. Red indicates that the genes act as predictors of an unfavorable prognosis, while blue indicates that they act as predictors of a favorable prognosis in The Cancer Genome Atlas (TCGA) training cohort. (b,c) LASSO penalized Cox regression analysis exhibiting the seven important hypoxia-driven genes.

Univariate Cox regression analysis identified 13 important hypoxia-driven DEGs as predictors of an unfavorable prognosis and three important hypoxia-driven DEGs as predictors of a favorable prognosis in TCGA training cohort (Figure 3(a)).

**Figure 4.**
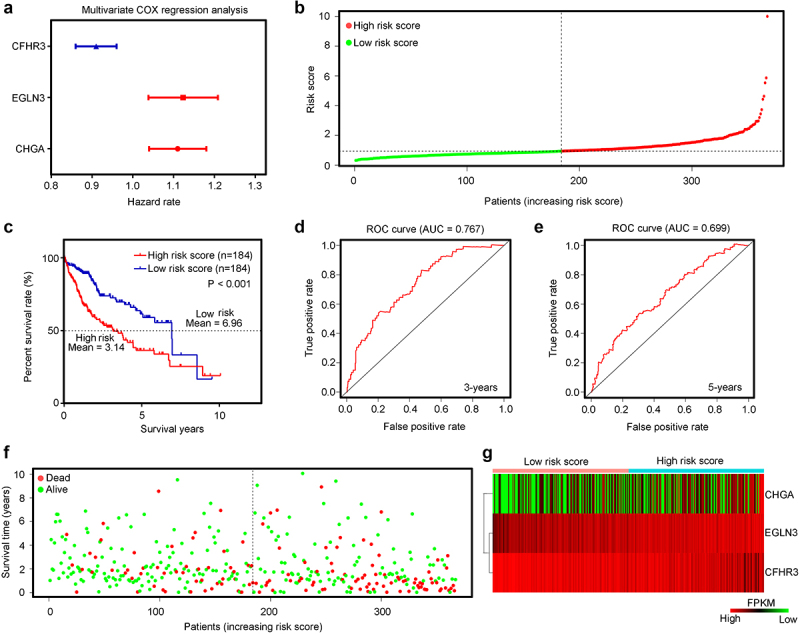
Construction and examination of the risk model in TCGA training cohort. (a) Multivariate COX regression analysis for egl-9 family hypoxia inducible factor 3 (*EGLN3*), chromogranin A (*CHGA*), and chromogranin A (*CFHR3*). Red indicates that the genes act as predictors of an unfavorable prognosis, while blue indicates that they act as predictors of a favorable prognosis in TCGA training cohort. The risk model was constructed by these three genes. (b) HCC samples in TCGA were divided into low-risk and high-risk groups. (c) Kaplan–Meier plot analysis of the overall survival rate in low-risk and high-risk patients with HCC in TCGA. (d, e) Receiver operating characteristic (ROC) curves showing the diagnostic value of the risk model for the 3-year and 5-year survival rates of patients with HCC in TCGA. (f) Survival time and status of each HCC patient in TCGA cohort. (g) Expression levels of CHGA, EGLN3 and CFHHR3 in HCC tissues from patients with high-risk and low-risk scores in TCGA.

LASSO-penalized Cox regression analysis was then used, and seven important hypoxia-driven DEGs, including *CHGA, EGLN3, PPFIA4, CXCL5, CRHBP, PFKP*, and *CFHR3*, were identified (Figure 3(b,c)). Stepwise multivariate Cox regression analysis was performed, and the results indicated that *CHGA, EGLN3*, and *CFHR3* also acted as significant predictors of risk (Figure 4(a)). Therefore, *CHGA, EGLN3*, and *CFHR3* were used to establish a predictive signature for patients with HCC in TCGA training cohort with a risk score of (0.101109253 × CHGA expression) + (0.116251865 × EGLN3 expression) + (–0.094009858 × CFHR3 expression). According to the median expression value of the risk score, patients in TCGA training cohort were divided into high- and low-risk groups (Figure 4(b)). The results demonstrated that Patients with HCC with a high-risk score in TCGA training cohort had a shorter overall survival rate (Figure 4(c)). ROC analysis revealed that the AUCs for predict the 3-year and 5-year survival rates were 0.767 and 0.699, respectively, in Patients with HCC in TCGA training cohort (Figure 4(d,e)). Moreover, Patients with HCC in TCGA with a high-risk score had more deaths (Figure 4(f)). Furthermore, heatmap analyses demonstrated that CHGA and EGLN3 were highly expressed in the HCC tissues of the high-hypoxia group in TCGA, while the expression of CFHR3 was reduced (Figure 4(g)).

### Verification of the prognostic risk model in the test cohort ICGC

**Figure 5.**
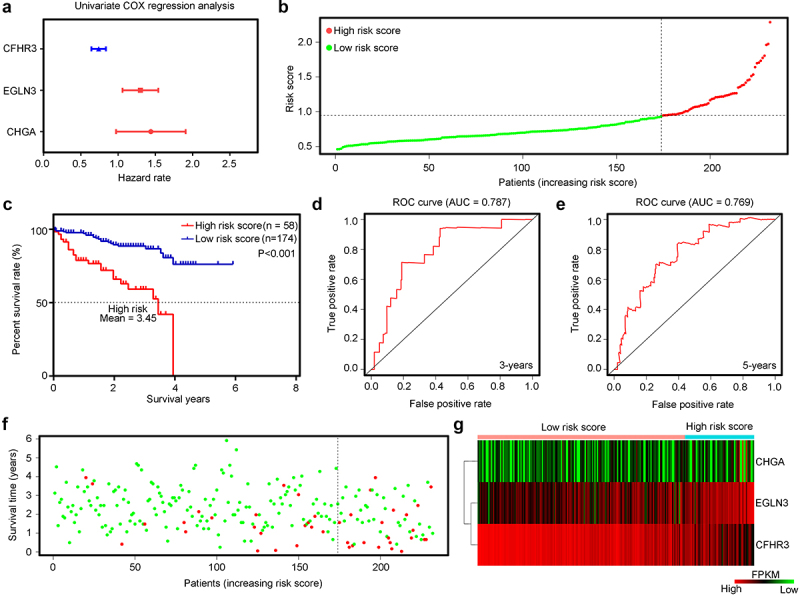
Examination of the risk model in the test cohort of the International Cancer Genome Consortium (ICGC). (a) Univariate Cox regression analysis for EGLN3, CHGA and CFHR3 in patients with HCC in the ICGC. Red indicates that the genes act as predictors of an unfavorable prognosis, while blue indicates that act as predictors of a favorable prognosis in the test cohort ICGC. (b) HCC samples in the ICGC were divided into low-risk and high-risk groups. (c) Kaplan–Meier plot analysis of the overall survival rate of low-risk and high-risk group patients with HCC in the ICGC. (d,e) ROC curves showing the diagnostic value of the risk model for the 3-year and 5-year survival rates of Patients with HCC in the ICGC. (f) Survival time and status of each HCC patient in the ICGC cohort. (g) Expression levels of CHGA, EGLN3, and CFHHR3 in HCC tissues from patients with high-risk and low-risk scores in the ICGC.

To test the applicability of the prognostic risk model, 232 HCC tissue samples from the ICGC database were used as the test cohort. The results demonstrated that CFHR3 was a predictor of a favorable prognosis in HCC tissues in ICGC, while EGLN3 and CHGA were predictors of an unfavorable prognosis (Figure 5(a)). According to the prognostic risk model constructed in TCGA training cohort and the medium-risk score, HCC tissues in the test cohort ICGC were also divided into high-risk and low-risk score groups (Figure 5(b)). The results indicated that Patients with HCC with a high-risk score also had a lower overall survival rate (Figure 5(c)). ROC analysis revealed that the AUC for predicting the 3-year and 5-year survival rates were 0.787 and 0.769 in Patients with HCC in the ICGC test cohort, respectively (Figure 5(d,e)). Consistent with the results in the training cohort, we found that the high-risk score group of patients with HCC in the test cohort ICGC had more deaths (Figure 5(f)). Furthermore, the expression of CHGA and EGLN3 was elevated in HCC tissues with a high-risk score in the test cohort ICGC, while the expression of CFHR3 was decreased (Figure 5(g)). Taken together, the prognostic risk model constructed using the three-gene signature (*CHGA, EGLN3*, and *CFHR3*) has the potential to provide valuable clinical utility for prognostic prediction in patients with HCC.

### GSEA analysis of the three gene signature

**Figure 6.**
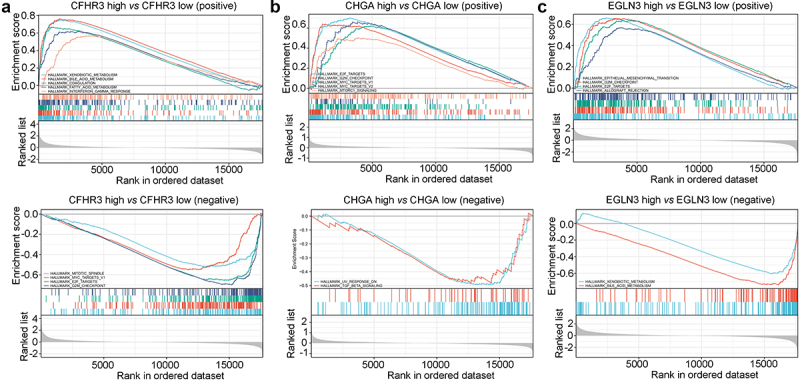
GSEA analysis of target genes in TCGA. GSEA of (a) *CFHR3*, (b) *CHGA*, (c) *EGLN3.*

HCC tissues in TCGA were divided into high expression and low expression groups according to the expression of CHGA, EGLN3, and CFHR3. The results indicated that CFHR3 expression was positively associated with the enrichment terms of ‘xenobiotic metabolism,’ ‘bile acid metabolism,’ ‘coagulation,’ ‘fatty acid metabolism,’ and ‘interferon gamma response,’ while the expression of CFHR3 was negatively associated with the enrichment terms of ‘mitotic spindle,’ ‘MYC target,’ ‘E2F target,’ and ‘G2/M checkpoint’ (Figure 6(a)). The expression of CHGA was positively associated with ‘E2F target,’ ‘G2/M checkpoint,’ ‘MYC target,’ and ‘MTORC1 signaling,’ and was negatively associated with the enrichment terms of ‘UV response DN’ and ‘TGF-β signaling’ (Figure 6(b)). The expression of EGLN3 was positively associated with the enrichment terms of ‘epithelial-mesenchymal transition,’ ‘G2/M checkpoint,’ ‘E2F targets,’ and ‘Allograft rejection,’ and was negatively associated with the enrichment terms of ‘xenobiotic metabolism’ and ‘bile acid metabolism’ (Figure 6(c)).

### Analysis of the diagnostic value of the three gene signature in serum specimens from patients with HCC

**Figure 7.**
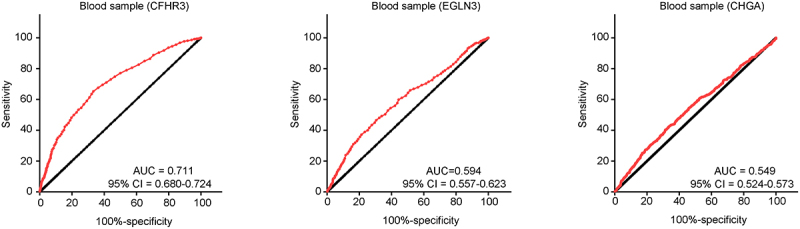
Diagnostic value of target genes (*CFHR3, EGLN3*, and *CHGA*) to distinguish the serum samples from patients with HCC and those from healthy controls.

GSE112679 contains 1,204 serum specimens from patients with HCC and 958 serum specimens from healthy individuals and was downloaded from the GEO database. The results of the ROC analysis indicated that CFHR3 had a remarkable diagnostic value (AUC = 0.711) in serum specimens for distinguishing Patients with HCC from healthy controls, whereas EGLN3 (AUC = 0.594) and CHGA (AUC = 0.549) had no diagnostic value (Figure 7). Therefore, we focused on CFHR3.

### Analysis of the expression model of CFHR3, EGLN3 and CHGA under hypoxia

**Figure 8.**
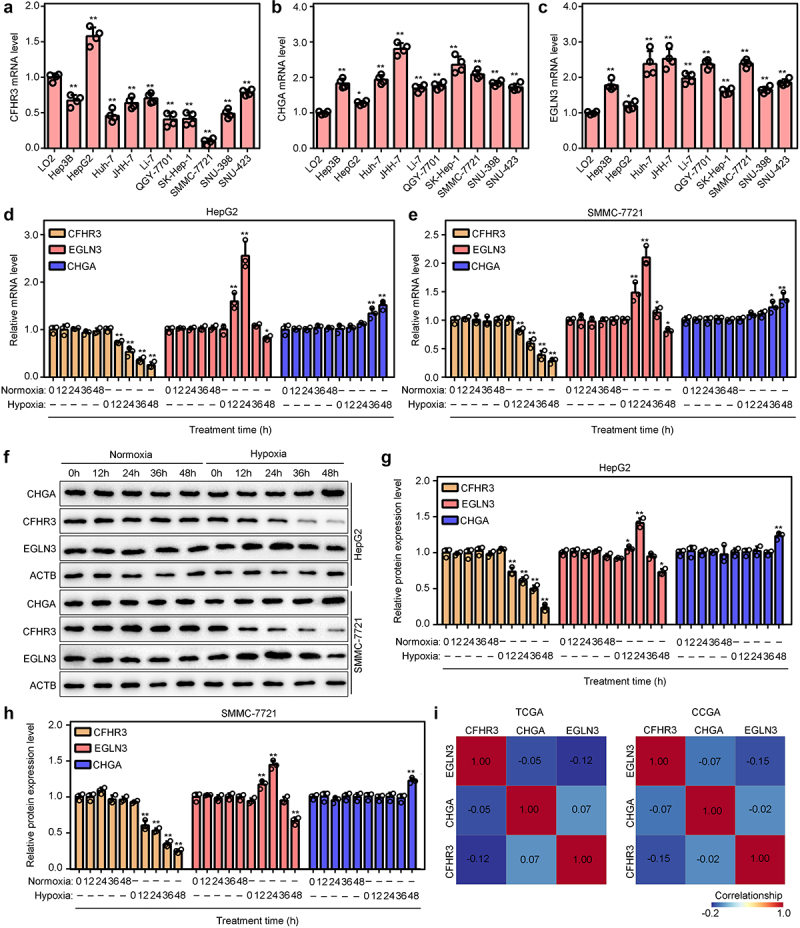
Analysis of the expression model of CFHR3, EGLN3 and CHGA under hypoxia. qRT-PCR was performed to detect the mRNA levels of CFHR3 (a), EGLN3 (b) and CHGA (c) in HCC cells lines. (d, e) qRT-PCR was performed to detect the mRNA levels OF CFHR3, EGLN3 and CHGA under normoxia and hypoxia for 0 h, 12 h, 24 h, 36 h and 48 h. (F, G and H) Western blot was performed to detect the protein levels OF CFHR3, EGLN3 and CHGA under normoxia and hypoxia for 0 h, 12 h, 24 h, 36 h and 48 h. (i) The co-expression relationship between CFHR3, EGLN3 and CHGA in HCC tissues in both TCGA and ICGC database.

We first detected the mRNA levels of CFHR3, EGLN3 and CHGA in HCC cell lines (Hep3B, Huh-7, JHH-7, Li-7, QGY-7701, SK-Hep-1, SNU-398, SNU-423, HepG2 and SMMC-7721) and normal hepatic epithelial cells (LO2). It was demonstrated that mRNA level of CFHR3 was decreased in most HCC cell lines compared with LO2, while it was overexpressed in HepG2 cells (Figure 8(a)). The expression of EGLN3 and CHGA was overexpressed in all HCC cell lines compared with those in LO2 cells (Figure 8(b,c)). We select HepG2 (high CFHR3, high EGLN3 and high CHGA) and SMMC-7721 cells (low CFHR3, high EGLN3 and high CHGA) cells as research model for further research. HepG2 and SMMC-7721 cells were then treated under normoxia and hypoxia. The mRNA level (Figure 8(d,e)) of CFHR3, EGLN3 and CHGA had no significant change under culturing in normoxia for 12 h, 24 h, 36 h and 48 h. The mRNA level of CFHR3 was significantly decreased under hypoxia for 12 h, 24 h, 36 h and 48 h. The mRNA level of EGLN3 was increased in 12 h and 24 h after culturing under hypoxia, while it was decreased in 48 h. The mRNA level of CHGA only slightly increased in 36 h and 48 h after culturing under hypoxia (Figure 8(d,e)). Moreover, the change trend of protein levels of CFHR3, EGLN3 and CHGA was consistent with mRNA levels (Figure 8(f-h)). Furthermore, we found that CFHR3, EGLN3 and CHGA had no co-expression relationship with each other in HCC tissues in both TCGA and ICGC database (Figure 8(i)). These results indicated that CFHR3, EGLN3 and CHGA had different expression model under hypoxia.

### Exploration of the biological functions of CFHR3 under hypoxia in vitro

**Figure 9.**
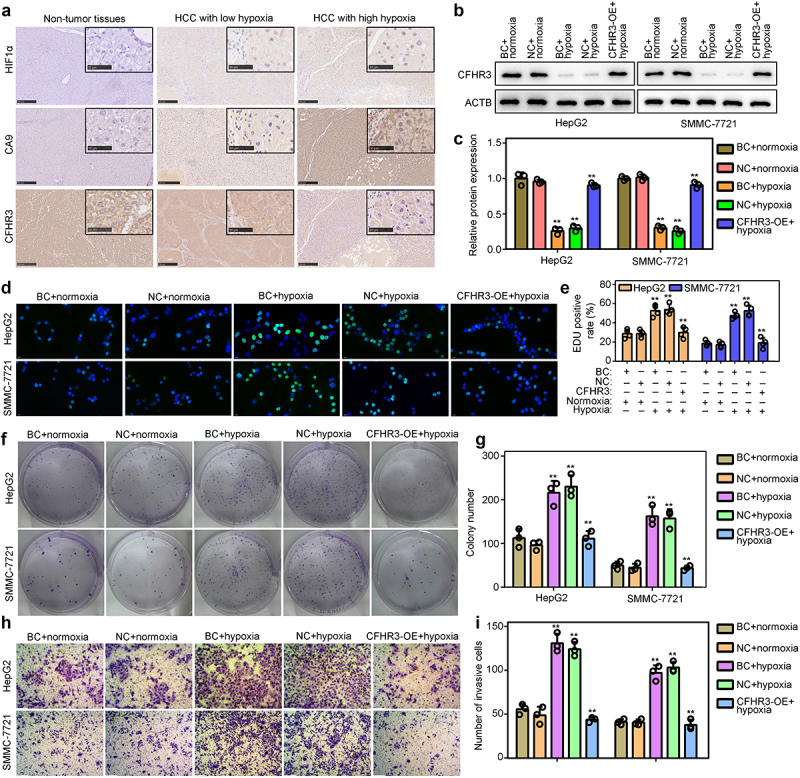
Exploration of the biological functions of CFHR3 in HCC cells *in vitro*. (a) Immunohistochemical staining demonstrates that CFHR3 expression is decreased in HCC tissues with high expression levels of hypoxia inducible factor 1 subunit alpha (HIF1A) and carbonic anhydrase 9 (CA9). (b-c) Western blot was used to detect the expression of CFHR3 in HCC cells in the blank control group (BC, without any treatment; cultured under normoxia and hypoxia), in the negative control group (NC, transfected with vector; cultured under normoxia and hypoxia) and in the CFHR3 overexpressing group (CFHR3-OE; cultured under hypoxia). (d-e) 5-ethynyl-2’-deoxyuridine (EdU) assays were used to detect the effects of CFHR3 on HCC cell proliferation under hypoxia. (f-g) Colony formation assays were used to detect the effects of CFHR3 on HCC cell colony formation under hypoxia. (h-i) Transwell assays were used to detect the effects of CFHR3 on HCC cell invasiveness under hypoxia. **P < 0.01.

We then determined the expression level of CFHR3 in 40 HCC tissues. HIF1A and CA9 were used as biomarkers of hypoxia, and immunohistochemical staining indicated that the expression of CFHR3 was reduced in HCC tissues with high hypoxia levels compared with adjacent normal tissues, while had no significant change in HCC tissues with low hypoxia levels (Figure 9(a)). We rescued the expression of CFHR3 in HepG2 and SMMC-7721 HCC cells cultured under hypoxic conditions (Figure 9(b,c)). EDU assays (Figure 9(d,e)) and colony formation assays (Figure 9(f,g)) demonstrated that hypoxia induced the increased proliferation of HepG2 and SMMC-7721 HCC cells, while the overexpression of CFHR3 blocked the stimulatory effects of hypoxia. Moreover, transwell assays indicated that overexpression of CFHR3 in HepG2 and SMMC-7721 HCC cells reversed the stimulative effects of hypoxia on cell invasion (Figure 9(h,i)). These results indicated that CFHR3 may be a key target for HCC therapy.

### Exploration of the biological functions of CFHR3 in vivo

**Figure 10.**
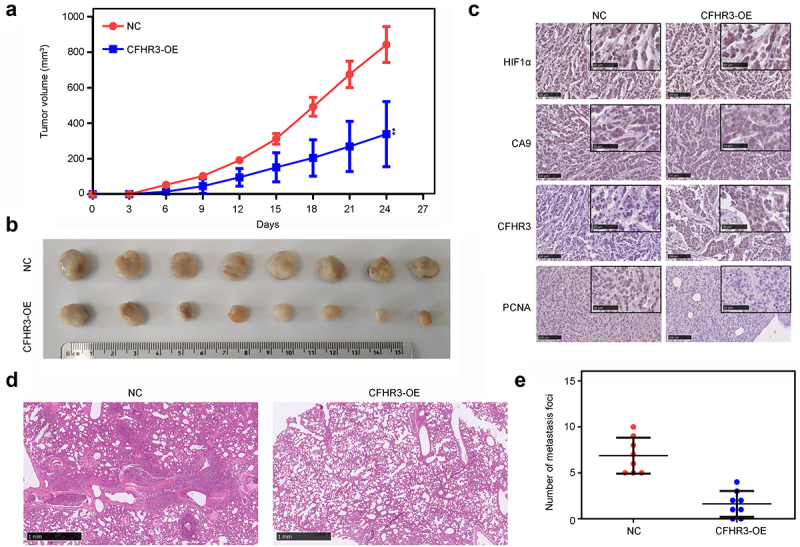
Exploration of the biological functions of CFHR3 in HCC cells *in vivo*. (a, b) The proliferation of HepG2 cells with CFHR3 overexpression and NC cells *in vivo*. (c) Expression levels of HIF1α, CA9, CFHR3, and proliferating cell nuclear antigen (PCNA) in tumor tissues derived from HepG2 cells with CFHR3 overexpression and NC cells. (d, e) Metastatic foci in the lungs from mice injected with SMMC-7721 cells with CFHR3 overexpression and NC cells. **P < 0.01.

Because a hypoxic microenvironment spontaneously occurs during tumor progression *in vivo*, we determined the effects of CFHR3 *in vivo*. Tumor tissues produced in mice from HepG2 cells overexpressing CFHR3 had a lower proliferation rate *in vivo* (Figure 10 (a.b)). Even though the expression of HIF1α and CA9 was the same in these two groups, tumor tissues derived from mice injected with HepG2 cells overexpressing CFHR3 had lower PCNA expression levels (Figure 10(c)). Similarly, SMMC-7721 HCC cells overexpressing CFHR3 showed a weaker ability to metastasize *in vivo* (Figure 10(d,e)).

## Discussion

The hypoxic microenvironment is an important factor that induces HCC cell proliferation and metastasis [25]. Therefore, the identification of hypoxia-driven gene expression patterns may help uncover the molecular mechanism of hypoxia responses in HCC, as well as provide potential biomarkers to diagnose HCC and identify effective therapeutic targets. In the present study, we were the first to construct a reliable prognostic signature based on three hypoxia-driven genes (*CFHR3, CHGA*, and *EGLN3*) and confirm its clinical utility in Patients with HCC. Furthermore, we are the first to indicate that CFHR3 can act as a diagnostic serum biomarker to distinguish Patients with HCC from healthy controls and is an effective target to block the stimulatory effects of hypoxia on HCC cells.

The solid tumor microenvironment has three well-known characteristics: hypoxia, nutrient deficiency and acidosis [26]. In recent years, hypoxia has been widely studied and is considered to be an important factor that promotes tumor progression. A series of onco-mRNAs, miRNAs, lncRNAs, and circRNAs driven by hypoxia have been identified in HCC [27]. Hypoxia-driven factors are highly expressed in HCC tissues and regulate cell proliferation, mobility, drug resistance, metabolic reprogramming, and angiogenesis. Similarly, a series of hypoxia-driven genes in HCC with prognostic value have been identified in previous studies. For example, aldolase A driven by hypoxia was found to be increased in HCC tissues, and a high expression level of aldolase A was associated with poor prognosis [28]. Hypoxia induces an increase in KIAA1199 expression in HCC, and a high expression level of KIAA1199 is positively associated with vascular invasion, TNM stage, and tumor size [29]. However, the prognostic value of these hypoxia-driven genes has only been verified in small cohorts, and their effectiveness has not been proven in larger cohorts. In previous studies [30,31], some prognostic models were constructed by known hypoxia associated genes and exhibited certain prognostic efficiency for HCC. In the current study, we first identified novel hypoxia-driven genes between HCC tissues with high and low hypoxia signature, and used the novel hypoxia-driven genes (EGLN3, CHGA and CFHR3) to construct a risk model, and it showed remarkable prognostic effectiveness in HCC in both TCGA and ICGC cohorts. These results indicate that the risk model may have a wide range of utilities.

A popular trend has been to use a noninvasive method to diagnose HCC [32]. Until now, serum AFPis the only ‘gold standard’ as a serum biomarker for the diagnosis of HCC, but not all HCC cells secrete AFP. A series of systematic reviews indicated that while AFP levels ≥20 ng/mL were set as cutoff values for diagnosing HCC, the sensitivity and specificity of AFP were 41–65% and 80–94%, respectively [33,34]. Therefore, identification of novel serum biomarkers may contribute to the diagnosis of HCC. Based on evidence that the risk model constructed by CFHR3, EGLN3, and CHGA has prognostic value, we considered whether it had a diagnostic value. Through analysis of a cohort of 1,204 serum specimens from Patients with HCC and 958 serum specimens from healthy individuals, we found that CFHR3 has a prominent diagnostic value with an AUC>0.7. Therefore, CFHR3 may be an important diagnostic serum biomarker.

EGLN3 is a cellular oxygen sensor that catalyzes, under normoxic conditions, the post-translational formation of 4-hydroxyproline in HIF1α proteins. Hydroxylated HIF1α proteins were then degraded by ubiquitination pathway [35]. Dysregulation of EGLN3 was observed in various cancers, however, it can act as both oncogene and suppressor in cancers [36,37]. Previous studies indicated that there are various posttranscriptional modifications in EGLN3; it was increased in mild hypoxia, and reduced in severe hypoxia [38]. Consistent with previous studies, we found EGLN3 was increased in short time, while reduced in long time after hypoxic treatment. CHGA is a member of the chromogranin/secretogranin family of neuroendocrine secretory proteins [39]. It is found in secretory vesicles of neurons and endocrine cells. CHGA was found in a series of cancers, and predicted poor outcome [40,41]. However, until now, its relationship with hypoxia was known limit. In the current study, we found that CHGA was slightly increased in long time after hypoxic treatment.

CFHR3 is a member of the human factor H protein family that can bind to heparin and may be involved in complement regulation [42]. Dysregulation of CFHR3 has been associated with a series of diseases, including age-related macular degeneration [43], atypical hemolytic uremic syndrome [44] and cancers [45]. Elevated expression of CFHR3 has been observed in gallbladder carcinomas compared with that in adjacent tissues [46]. Loss of CFHR3 function in gliomas induces cisplatin resistance [47]. Similarly, previous studies have indicated that overexpression of CFHR3 inhibits the proliferation of HCC cells *in vitro* [48]. Consistent with previous studies, the biological experiments performed in the present study also indicated that CFHR3 plays a suppressive role in HCC. Furthermore, we provide the first evidence that CFHR3 is linked to the hypoxic environment and malignant biological properties of HCC cells. CFHR3 expression was reduced in HCC tissues with a high hypoxia level and in HCC cells cultured under hypoxic conditions. Hypoxia promoted the proliferation and mobility of HCC cells, whereas overexpression of CFHR3 blocked the stimulatory effects of hypoxia. Additionally, we found that CFHR3 had the potential to suppress HCC cell proliferation and metastasis *in vivo*. These results indicate that CFHR3 may be an effective target for blocking the stimulatory effects of hypoxia.

However, some limitations were also existed in our present study. The molecular mechanism of CFHR3 under hypoxia was still known limited. Moreover, more experiments need to be performed to determine the regulatory mechanisms of EGLN3 under hypoxia.

## Conclusion

In conclusion, the risk model constructed using three hypoxia-driven genes (*CFHR3, CHGA*, and *EGLN3*) is valuable for the prognostic prediction of patients with HCC. CFHR3 also has diagnostic value and acts as a suppressor in HCC cells by blocking the stimulatory effects of the hypoxic environment.

## Supplementary Material

Supplemental Material

## Data Availability

The RNA-seq data of HCC used for the training and test cohorts were downloaded from TCGA (https://portal.gdc.cancer.gov) and ICGC (https://dcc.icgc.org/). The experimental datasets used and analyzed in this study are available from the corresponding authors upon reasonable request.
