# Supplementary material for: A novel hypoxia-driven gene signature that can predict the prognosis of hepatocellular carcinoma
Source: Bioengineered. 2022 May 13;13(5):12193–210. doi: 10.1080/21655979.2022.2073943 (PMC9276011; doi:10.1080/21655979.2022.2073943)
Supplement: Supplemental Material [file KBIE_A_2073943_SM3152.docx]

| Group | ID |
| --- | --- |
| High hypoxia | TCGA-2Y-A9H1-01 TCGA-ED-A97K-01 TCGA-BC-A3KG-01 TCGA-ED-A7XP-01 TCGA-CC-A7IK-01 TCGA-ED-A627-01 TCGA-DD-AAC9-01 TCGA-BC-A10X-01 TCGA-K7-A5RG-01 TCGA-ZP-A9D2-01 TCGA-G3-A3CJ-01 TCGA-MI-A75G-01 TCGA-BC-A3KF-01 TCGA-2Y-A9H4-01 TCGA-G3-A25Z-01 TCGA-ES-A2HS-01 TCGA-DD-A73F-01 TCGA-BC-A110-01 TCGA-UB-A7MA-01 TCGA-DD-AAVQ-01 TCGA-CC-5260-01 TCGA-DD-AADF-01 TCGA-DD-A1EI-01 TCGA-DD-AADQ-01 TCGA-KR-A7K0-01 TCGA-BC-A216-01 TCGA-5R-AA1D-01 TCGA-DD-A118-01 TCGA-RC-A6M5-01 TCGA-RC-A7S9-01 TCGA-CC-A7IF-01 TCGA-DD-A1EE-01 TCGA-G3-AAV5-01 TCGA-2Y-A9H2-01 TCGA-DD-AACI-01 TCGA-ZS-A9CD-01 TCGA-G3-A5SI-01 TCGA-2Y-A9H3-01 TCGA-DD-A73B-01 TCGA-G3-AAUZ-01 TCGA-G3-A5SM-01 TCGA-UB-A7ME-01 TCGA-DD-AAD0-01 TCGA-G3-A25Y-01 TCGA-PD-A5DF-01 TCGA-DD-AAEE-01 TCGA-2Y-A9H8-01 TCGA-ZP-A9CZ-01 TCGA-BW-A5NO-01 TCGA-FV-A2QQ-01 TCGA-BC-A112-01 TCGA-O8-A75V-01 TCGA-ED-A66X-01 TCGA-BD-A3EP-01 TCGA-G3-A25V-01 TCGA-RC-A7SF-01 TCGA-2Y-A9GS-01 TCGA-WQ-AB4B-01 TCGA-RC-A7SB-01 TCGA-DD-AACC-01 TCGA-DD-A113-01 TCGA-K7-A6G5-01 TCGA-DD-AADR-01 TCGA-BC-A217-01 TCGA-CC-A3MB-01 TCGA-CC-A9FS-01 TCGA-DD-AADM-01 TCGA-HP-A5MZ-01 TCGA-ES-A2HT-01 TCGA-CC-A123-01 TCGA-FV-A3I1-01 TCGA-2Y-A9GW-01 TCGA-DD-AADB-01 TCGA-FV-A495-01 TCGA-DD-AAC8-01 TCGA-G3-AAV3-01 TCGA-DD-AAVP-01 TCGA-DD-AAE9-01 TCGA-DD-AAVU-01 TCGA-DD-A114-01 TCGA-MI-A75E-01 TCGA-5C-A9VG-01 TCGA-DD-AADK-01 TCGA-DD-A1EC-01 TCGA-GJ-A3OU-01 TCGA-BC-A8YO-01 TCGA-G3-A25S-01 TCGA-DD-AAVV-01 TCGA-DD-AAVR-01 TCGA-DD-A1EG-01 TCGA-DD-A116-01 TCGA-CC-A5UD-01 TCGA-DD-AAEI-01 TCGA-UB-A7MD-01 TCGA-DD-AAEG-01 TCGA-DD-AAE0-01 TCGA-G3-A7M7-01 TCGA-DD-AAD2-01 TCGA-DD-A119-01 TCGA-FV-A4ZQ-01 TCGA-DD-A39X-01 TCGA-CC-A3MA-01 TCGA-RG-A7D4-01 TCGA-MI-A75I-01 TCGA-DD-A1EJ-01 TCGA-DD-A3A1-01 TCGA-CC-A9FW-01 TCGA-EP-A3RK-01 TCGA-DD-A1EK-01 TCGA-EP-A2KA-01 TCGA-DD-A4NH-01 TCGA-MI-A75C-01 TCGA-CC-A3MC-01 TCGA-DD-AACX-01 TCGA-ED-A7XO-01 TCGA-WX-AA47-01 TCGA-CC-A5UC-01 TCGA-BC-A10W-01 TCGA-2Y-A9HA-01 TCGA-DD-A3A7-01 TCGA-CC-5262-01 TCGA-DD-A39V-01 TCGA-CC-5258-01 TCGA-G3-A5SJ-01 TCGA-DD-AACD-01 TCGA-DD-AADC-01 TCGA-CC-5259-01 TCGA-UB-A7MF-01 TCGA-BC-4072-01 TCGA-DD-AADO-01 TCGA-ED-A8O6-01 TCGA-EP-A3JL-01 TCGA-DD-AACG-01 TCGA-CC-A8HU-01 TCGA-DD-A73G-01 TCGA-G3-AAV6-01 TCGA-DD-AA3A-01 TCGA-ED-A7PX-01 TCGA-CC-A5UE-01 TCGA-DD-A4NR-01 TCGA-WQ-A9G7-01 TCGA-FV-A3I0-01 TCGA-DD-A4NA-01 TCGA-KR-A7K7-01 TCGA-DD-AACB-01 TCGA-XR-A8TD-01 TCGA-MR-A8JO-01 TCGA-DD-AADN-01 TCGA-DD-AACH-01 TCGA-K7-AAU7-01 TCGA-5C-AAPD-01 TCGA-BC-A10Q-01 TCGA-DD-AACZ-01 TCGA-2Y-A9H0-01 TCGA-CC-A7IG-01 TCGA-DD-A3A3-01 TCGA-CC-A1HT-01 TCGA-CC-5264-01 TCGA-DD-AAD5-01 TCGA-CC-A7IE-01 TCGA-FV-A4ZP-01 TCGA-BC-A10U-01 TCGA-2Y-A9GY-01 TCGA-DD-A1EL-01 TCGA-DD-AACP-01 TCGA-YA-A8S7-01 TCGA-BC-4073-01 TCGA-CC-A8HT-01 TCGA-ED-A459-01 TCGA-G3-A25T-01 TCGA-BC-A69H-01 TCGA-GJ-A6C0-01 TCGA-DD-AACL-01 TCGA-G3-A25X-01 TCGA-CC-5263-01 TCGA-CC-A7IJ-01 TCGA-RC-A6M6-01 TCGA-DD-A4NQ-01 TCGA-ED-A7PZ-01 TCGA-DD-A39Y-01 TCGA-CC-A3M9-01 TCGA-G3-AAV7-01 TCGA-G3-A7M9-01 TCGA-CC-A7II-01 |
| Low hypoxia | TCGA-DD-AADY-01 TCGA-G3-A6UC-01 TCGA-DD-AADA-01 TCGA-2Y-A9GV-01 TCGA-DD-A73D-01 TCGA-DD-A39W-01 TCGA-ZP-A9D4-01 TCGA-DD-A4NF-01 TCGA-DD-A3A2-01 TCGA-DD-AAVY-01 TCGA-DD-A73E-01 TCGA-DD-A73C-01 TCGA-DD-AACE-01 TCGA-DD-A4NI-01 TCGA-G3-AAV0-01 TCGA-EP-A26S-01 TCGA-DD-A1EB-01 TCGA-DD-A11B-01 TCGA-ED-A7PY-01 TCGA-3K-AAZ8-01 TCGA-DD-A4NK-01 TCGA-DD-A4NP-01 TCGA-XR-A8TE-01 TCGA-HP-A5N0-01 TCGA-DD-AACN-01 TCGA-DD-A11D-01 TCGA-ZS-A9CF-02 TCGA-DD-A1EH-01 TCGA-WX-AA46-01 TCGA-EP-A12J-01 TCGA-MR-A520-01 TCGA-DD-AAEB-01 TCGA-G3-A3CG-01 TCGA-DD-AADG-01 TCGA-DD-A4NV-01 TCGA-DD-A4NL-01 TCGA-2Y-A9GZ-01 TCGA-DD-AAW3-01 TCGA-DD-AACQ-01 TCGA-DD-A73A-01 TCGA-DD-AAE1-01 TCGA-RC-A7SH-01 TCGA-G3-AAV2-01 TCGA-CC-5261-01 TCGA-WJ-A86L-01 TCGA-DD-AAW1-01 TCGA-G3-A7M8-01 TCGA-2Y-A9H6-01 TCGA-G3-A5SK-01 TCGA-FV-A2QR-01 TCGA-UB-AA0V-01 TCGA-DD-AACW-01 TCGA-G3-A3CI-01 TCGA-2Y-A9HB-01 TCGA-DD-AADU-01 TCGA-ZP-A9CY-01 TCGA-ZP-A9CV-01 TCGA-ZP-A9D1-01 TCGA-DD-AAE7-01 TCGA-2Y-A9H9-01 TCGA-DD-A3A4-01 TCGA-2Y-A9GT-01 TCGA-ZS-A9CE-01 TCGA-DD-A1ED-01 TCGA-LG-A9QD-01 TCGA-DD-AAEK-01 TCGA-DD-AACA-02 TCGA-G3-A25U-01 TCGA-ED-A4XI-01 TCGA-LG-A9QC-01 TCGA-FV-A23B-01 TCGA-DD-AACY-01 TCGA-K7-A5RF-01 TCGA-DD-A1EA-01 TCGA-DD-AAD3-01 TCGA-BC-A5W4-01 TCGA-DD-AACS-01 TCGA-BC-A10Y-01 TCGA-DD-AAEH-01 TCGA-ED-A8O5-01 TCGA-ZP-A9D0-01 TCGA-CC-A7IH-01 TCGA-ED-A66Y-01 TCGA-XR-A8TC-01 TCGA-DD-A1EF-01 TCGA-DD-A115-01 TCGA-XR-A8TF-01 TCGA-G3-A3CK-01 TCGA-DD-AAD8-01 TCGA-ZS-A9CF-01 TCGA-FV-A496-01 TCGA-G3-A7M5-01 TCGA-UB-A7MC-01 TCGA-DD-AAVS-01 TCGA-2Y-A9H5-01 TCGA-2Y-A9H7-01 TCGA-CC-A8HS-01 TCGA-5R-AA1C-01 TCGA-MI-A75H-01 TCGA-DD-AAW2-01 TCGA-DD-AADW-01 TCGA-DD-A3A8-01 TCGA-DD-A4NS-01 TCGA-DD-AAED-01 TCGA-DD-AACV-01 TCGA-DD-AACU-01 TCGA-BD-A2L6-01 TCGA-RC-A7SK-01 TCGA-G3-A3CH-01 TCGA-EP-A2KC-01 TCGA-ZS-A9CG-01 TCGA-DD-AAD6-01 TCGA-DD-A4NO-01 TCGA-FV-A3R3-01 TCGA-DD-AAE3-01 TCGA-DD-AAVZ-01 TCGA-LG-A6GG-01 TCGA-DD-AACA-01 TCGA-DD-A4NG-01 TCGA-DD-A3A6-01 TCGA-DD-AACO-01 TCGA-DD-AACJ-01 TCGA-BC-A10Z-01 TCGA-DD-AACT-01 TCGA-2Y-A9GX-01 TCGA-EP-A2KB-01 TCGA-KR-A7K2-01 TCGA-T1-A6J8-01 TCGA-DD-AADJ-01 TCGA-DD-AAVX-01 TCGA-DD-AAD1-01 TCGA-ED-A5KG-01 TCGA-BC-A10R-01 TCGA-WX-AA44-01 TCGA-UB-AA0U-01 TCGA-DD-AADP-01 TCGA-DD-AADI-01 TCGA-G3-A5SL-01 TCGA-DD-AAE2-01 TCGA-DD-AAVW-01 TCGA-NI-A4U2-01 TCGA-ED-A82E-01 TCGA-DD-A3A9-01 TCGA-BC-A69I-01 TCGA-DD-AADS-01 TCGA-DD-AAW0-01 TCGA-G3-A7M6-01 TCGA-CC-A7IL-01 TCGA-DD-AADD-01 TCGA-G3-AAV4-01 TCGA-DD-A11C-01 TCGA-DD-AAE6-01 TCGA-DD-A4NB-01 TCGA-UB-A7MB-01 TCGA-5R-AAAM-01 TCGA-DD-A39Z-01 TCGA-RC-A6M4-01 TCGA-DD-AAEA-01 TCGA-XR-A8TG-01 TCGA-QA-A7B7-01 TCGA-2Y-A9GU-01 TCGA-NI-A8LF-01 TCGA-DD-A4NE-01 TCGA-DD-AAE4-01 TCGA-CC-A8HV-01 TCGA-GJ-A9DB-01 TCGA-G3-AAV1-01 TCGA-DD-AADL-01 TCGA-DD-A4ND-01 TCGA-DD-AADV-01 TCGA-FV-A3R2-01 TCGA-4R-AA8I-01 TCGA-5C-A9VH-01 TCGA-DD-A3A5-01 TCGA-BD-A3ER-01 TCGA-BC-A10T-01 TCGA-DD-AACF-01 TCGA-FV-A2QR-11 TCGA-BC-A10S-01 TCGA-DD-AACK-01 TCGA-DD-A4NN-01 TCGA-DD-A4NJ-01 TCGA-DD-A11A-01 TCGA-KR-A7K8-01 |
|  |  |
